# Deep Learning for Feature Extraction in Remote Sensing: A Case-Study of Aerial Scene Classification

**DOI:** 10.3390/s20143906

**Published:** 2020-07-14

**Authors:** Biserka Petrovska, Eftim Zdravevski, Petre Lameski, Roberto Corizzo, Ivan Štajduhar, Jonatan Lerga

**Affiliations:** 1 Ministry of Defense of Republic of North Macedonia, 1000 Skopje, North Macedonia; 2 Faculty of Computer Science and Engineering, Saints Cyril and Methodius University, 1000 Skopje, North Macedonia; eftim.zdravevski@finki.ukim.mk (E.Z.); petre.lameski@finki.ukim.mk (P.L.); 3 Department of Computer Science, University of Bari Aldo Moro, 70125 Bari, Italy; roberto.corizzo@uniba.it; 4 Department of Computer Science, American University, Washington, DC 20016, USA; 5 Faculty of Engineering, University of Rijeka, 51000 Rijeka, Croatia; istajduh@riteh.hr; 6 Center for Artificial Intelligence and Cybersecurity, University of Rijeka, Radmile Matejcic 2, 51000 Rijeka, Croatia

**Keywords:** remote sensing, convolutional neural network (CNN), feature extraction, feature fusion

## Abstract

Scene classification relying on images is essential in many systems and applications related to remote sensing. The scientific interest in scene classification from remotely collected images is increasing, and many datasets and algorithms are being developed. The introduction of convolutional neural networks (CNN) and other deep learning techniques contributed to vast improvements in the accuracy of image scene classification in such systems. To classify the scene from areal images, we used a two-stream deep architecture. We performed the first part of the classification, the feature extraction, using pre-trained CNN that extracts deep features of aerial images from different network layers: the average pooling layer or some of the previous convolutional layers. Next, we applied feature concatenation on extracted features from various neural networks, after dimensionality reduction was performed on enormous feature vectors. We experimented extensively with different CNN architectures, to get optimal results. Finally, we used the Support Vector Machine (SVM) for the classification of the concatenated features. The competitiveness of the examined technique was evaluated on two real-world datasets: UC Merced and WHU-RS. The obtained classification accuracies demonstrate that the considered method has competitive results compared to other cutting-edge techniques.

## 1. Related Work

Scene classification is one of the main tasks in aerial image understanding. In this process, a semantic label is assigned to images collected from remote locations [1,2]. Scene classification is also known as image classification when it comes to remote sensing datasets, and these two expressions are equally used throughout our paper. Remote sensing classification has found its usage in many areas: battlefields, traffic control, and disaster observation [3,4]. Remote sensing images are a composition of scene information and contain an enormous feature space describing the texture. Scene composition of remote sensing images are often compound, and because of this, it might be challenging to get the semantic label right from the image data [5,6]. This is the reason for developing numerous scene classification techniques. In general, they can belong to one of the following categories: methods that utilize low-level image features [7,8,9,10,11,12,13,14,15,16,17,18,19,20], methods using mid-level image representation [21,22,23,24,25,26,27,28,29,30,31,32,33,34,35], and methods using high-level image features [36,37,38,39,40,41,42,43,44,45]. In continuation, a brief literature review of extracted feature-based classification methods is given.

With methods using low-level visual features, aerial scenes are classified from low-level visual descriptors: spectral, textural, structural, and so on. The local fluctuation of structures in remote sensing images is modeled by Scale Invariant Feature Transform (SIFT) [7] as a local structure descriptor. Other descriptors utilize statistical and global distributions of specific image characteristics, like color [12] and texture data [8,9]. In [10], the authors used the IKONOS dataset to compare SIFT and Gabor texture features. In [11], authors compare various color and texture descriptors such as color histograms and local binary pattern (LBP) [13] descriptors. Authors in [14] use six various types of descriptors: SIFT, radiometric features, Grey Level Co-Occurrence Matrix (GLCM), Gaussian wavelet features [15], shape features [16], and Gabor filters, and make a combination of them in compound-feature figures with different spatial resolutions for remote sensing. In [17], the authors use Gist and SIFT descriptors. Other descriptors used in the literature are the orientation difference descriptor [18], and the Enhanced Gabor Texture Descriptor (EGTD) [19]. In [20], authors propose completed local binary patterns with multi-scales (MS-CLBP) for remote sensing image classification, and obtained cutting-edge results compared to other low-level methods.

The mid-level visual representation methods are another group of methods used for scene representation, which attempt to represent scenes with the statistical representation of high-degree locally extracted image features. They first perform local image feature extraction from local patches, using descriptors such as color histograms or SIFT. Then these features are encoded for composing a mid-level representation for remote sensing images. A commonly used mid-level method is bag-of-visual-words (BoVW) [21]. This method first describes local scene pieces using SIFT [7] descriptors, and afterward learns a vocabulary of visual words, e.g., utilizing k-means clustering. The vocabulary is called a visual dictionary or visual codebook. This mid-level technique and its variations are often suitable for representing aerial scenes. In remote sensing image classification tasks, the basic BoVW method can be used with different local descriptors [22]: SIFT, LBP, color histogram, GIST. Multiple features can be utilized for aerial scene classification, including hierarchical classification methods and normalization with Extreme Value Theory (EVT) [23]. Another approach is to apply a sparse coding method [24] where structural, spectral, and textural features are extracted and encoded. The state-of-the-art performance can be achieved by performing dimensionality reduction with Principal Component Analysis (PCA), before concatenating multi-features, or with methods such as the Improved Fisher Vector (IFK) [25] and Vectors of Locally Aggregated Tensors (VLAT) [26]. The methods mentioned earlier do not incorporate the spatial distribution of visual words, which, according to some authors, is important. Namely, [27] proposes the spatial pyramid co-occurrence kernel (SPCK), which, despite the basic BoVW model, integrates the absolute and relative spatial data. This method incorporates principles of spatial pyramid match kernel (SPM) [28] and spatial co-occurrence kernel (SCK) [21]. In [29], the authors develop a model to integrate absolute and relative spatial connections of local low-level representations into a pyramid-of-spatial-relations (PSR) model. In addition to BoVW, there are mid-level classification models that take into account the semantic connection between low-level local visual words for image representation. CIELab color moments [31] are used with Latent Dirichlet Allocation (LDA) [30] and in [20]. GLCM [32] and edge orientation histogram (EOH) [33] are other ways of extracting different information in the LDA model. The probabilistic Latent Semantic Analysis (pLSA) technique [35] is adopted in [34].

The third group of techniques for image classification relay on high-level vision information. There are a lot of computer vision assignments that can be successfully solved using deep learning methods: image classification, object recognition, and image retrieval. Compared to the methods exposed previously in this section, high-level methods can obtain more abstract and discriminative semantic representations, which results in the achievement of better classification performance. Feature extraction with convolutional neural networks (CNNs), pre-trained on massive datasets [36], accomplishes great performance for aerial scene classification [5]. There are many freely available pre-trained deep CNN architectures: ResNet, DenseNet, Inception, Xception, and so on. The classification accuracy of remote sensing images can be increased with a multiscale input strategy for multi-view CNN learning, similar to what was shown for GoogleNet in [6]. Multi-ratio dense CNN features from the final convolutional layer are extracted in [37], and afterward encoded with BoVW [38], Vector of Locally Aggregated Descriptors (VLAD) [39] and Improved Fisher Kernel (IFK) [25] to form the final scene presentation. Global features are extracted by Nogueira et al. [40] from the CNN models, and they are fed to a classifier. This method has shown the effectiveness of transfer learning from object classification models. In all of these examples, the global or local extracted feature representations were acquired from CNNs pre-trained on datasets with natural images. Those features were utilized for the classification of remote sensing images. Contrary to the deep learning methods described above, an alternative is to optimize CNN from scratch, i.e., from randomly set parameter values. However, as reported in [40], using the UC-Merced dataset [21] and the WHU-RS19 dataset [41] for full training of CNNs like CaffeNet [42] or GoogLeNet [43] shows poor classification performance. The reason for the poor performance and poor generalization is that large CNN architectures have a huge number of parameters, and model training from aerial scene datasets with up to thousands of images inevitably gets stuck in local minima. However, training CNNs from scratch using larger datasets, such as AID [44] and NWPU-RESISC45 [45], has shown good results.

### Introduction and Contributions 

In our article, we use a remote sensing image classification architecture that leverages pre-trained CNN models that are able to classify high-resolution aerial images. Our approach attempts to constitute better features for aerial scenes from neural networks’ activations. The CNNs which are included in our experiments are: ResNet50 [46], InceptionV3 [47], Xception [48], and DenseNet121 [49]. The models are fully trained on the ImageNet [36] dataset. These CNNs perform feature extraction by removing some of the layers of the original pre-trained network. Pre-trained CNNs have a complex architecture with tens of layers, and feature extraction is made from various layers, as in [37]. We use activations from the average pooling layer, last convolutional layer, and from some of the intermediate convolutional layers over the entire image, in order to obtain feature representations of the scene. By doing this, we get a convolutional feature vector with significant dimensionality. For this reason, feature dimensionality reduction methods are utilized prior to concatenating these features with the features extracted from average pooling layers. Following the feature extraction and feature fusion, there is a need for a classifier to get semantic labels of aerial images. Our article proposes two widely used linear classifiers—linear SVM and Logistic Regression Classifier (LRC)—to process the extracted features and classify the scenes. 

The used method generates excellent features for remote sensing scene classification. These features are obtained with modifications of the pre-trained CNN models. The achieved classification performance is on par with the state-of-the-art approaches.

The main contributions of this article are recapped as follows:

- Here, the last or intermediate CNN average pooling layers and convolutional layers are combined to generate image scene features.
- We compare pre-trained CNN models InceptionV3 [47], ResNet50 [46], Xception [48], and DenseNet121 for image features used for image scene classification.
- We present a technique for feature extraction utilizing pre-trained neural networks and perform dimensionality reduction of the dense CNN activations from the convolutional layers (either the last one or one of the intermediate convolutional layers) using the PCA. Afterward, feature fusion of the convolutional layer activations and average pooling layers activations is evaluated, based on the performance of linear classifiers Linear SVM and LRC.
- The examined technique is compared to the existing methods on two publicly available remote sensing datasets, providing a baseline for aerial scene classification with deep learning methods.

Datasets used are the UC-Merced dataset and the WHU-RS dataset. The reason for choosing these two remote sensing datasets lies in the fact that they are commonly used, so it was convenient to compare our experimental results with the achieved classification accuracies by other authors in the related articles. 

The remainder of this article is organized as follows. In Section 2, the methodologies used for feature extraction and classification are presented, and how they were empirically evaluated is described. Experimental results obtained from the examined remote sensing images classification technique are presented in Section 3. Several factors have an impact on our method’s results, and they are discussed in Section 4. We conclude and summarize the paper in Section 5.

## 2. Materials and Methods

**Figure 1:**
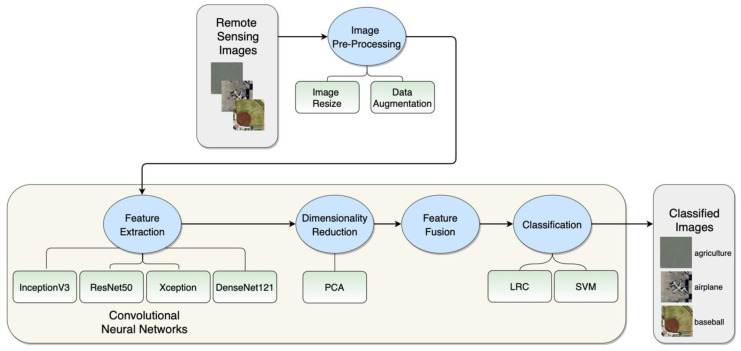
Workflow of the proposed method.

This section of the article briefly describes all pre-trained CNNs used for feature extraction: InceptionV3, ResNet50, Xception, and DenseNet121. These model architectures were used for feature extraction because they exhibited superb performance in image classification on the ImageNet dataset. Following that, we introduce the linear classifiers used for remote sensing images classification: LRC and SVM, as well as the PCA that was used for dimensionality reduction. We observed similar modeling techniques being used in related work [50]. Next, we describe the two publicly available datasets, which were utilized in our study: the UC-Merced and the WHU-RS19 dataset. We use these datasets to evaluate our approaches and compare them with related work. The section is closed with the description of the experimental setup and the evaluation metrics used. The general workflow of the proposed method and its phases are represented in Figure 1. 

### 2.1. Inception

**Figure 2:**
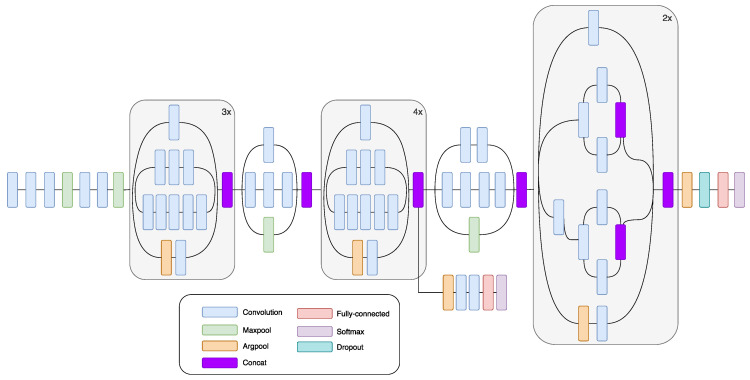
Schematic drawing of the InceptionV3 convolutional neural network (CNN) [51].

GoogLeNet is a CNN that won the ILSVRC-2014 contest in part for classification and detection tracks. GoogLeNet is a non-sequential CNN. It can increase its depth and width without causing computational strain [43]. GoogLeNet uses the so-called “Inception module.” It comes from the idea that multiple connections between layers lead to redundancy, because of the correlation between them. “Inception module” is a CNN itself. “Inception module” consists of 22 layers and processes its input in a parallel workflow. Within its intermediate layers, several auxiliary classifiers are included. Auxiliary classifiers are inserted to boost the discrimination capability in the lower layers. This module can use convolutional and pooling operations in each layer. For example, in AlexNet and VGG, each level uses either a convolutional or a pooling operation. The main characteristic of the module is that filters with different sizes are used in the same layer. This leads to a different size of the extracted patterns, as well as more exhaustive information. The bottleneck layer, which is a 1 × 1 convolutional layer, has a twofold function: to simplify the computations and to lower the CNN number of parameters. Besides the 1 × 1 convolutional layers, inception modules contain larger kernel convolutional filters, 3 × 3 and 5 × 5. In order to reduce the number of parameters at each level, 1 × 1 convolutional layers precede 3 × 3 and 5 × 5 convolutional layers. After 1 × 1 convolutional layers, ReLU is used, and the goal of this operation is to increase non-linearity and to deepen the network. In this network, there are no fully connected layers; the average pooling layer is used instead. The absence of fully connected layers decreases the number of parameters. The InceptionV3 network has fewer parameters compared to AlexNet and VGG and yet is able to learn deeper presentations of features [48]. Figure 2 shows a diagram of InceptionV3 CNN.

### 2.2. ResNet 

**Figure 3:**
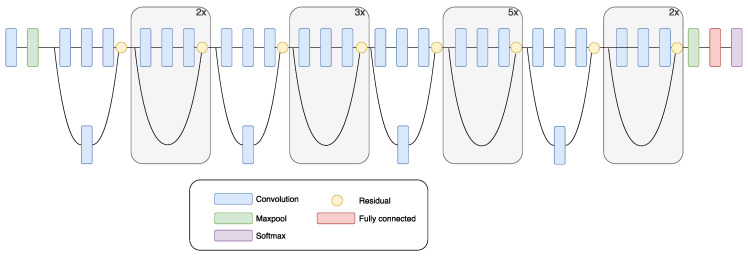
Schematic drawing of the ResNet CNN [51].

ResNet won the classification task of the ILSVRC-2015 contest. ResNet is a very deep CNN which consists of 152 layers [46]. Two main issues are related to difficulties in training deep architectures: high training error and the vanishing gradient problem. The problem with vanishing gradient causes learning inefficiency in network training. This inefficiency is present at the lower layers during backpropagation. ResNet solves the problem of vanishing gradient with an application of residual module. The deep learning residual module has a shortcut between the input and the output. The residual model has its mapping, which is fitted during the training phase by each layer in the module [46]. Network training is simpler on the residual map, compared to the underlying original network structure. ResNet contains mostly 3 × 3 convolutional filters. This property makes it similar to the VGG model. However, ResNet has fewer filters compared to the VGG network and is, therefore, simpler [46]. Figure 3 shows a schematic drawing of ResNet. 

### 2.3. Xception

**Figure 4:**
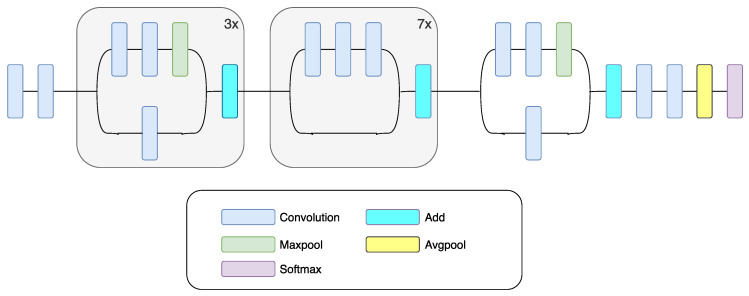
Schematic drawing of the Xception CNN [51].

Another deep network that is similar to GoogLeNet is Xception. In the Xception, depth-wise separable convolutional layers are used instead of the inception module [48]. The architecture of the Xception model is based on two types of convolutional layers: a depth-wise convolutional layer [52] and a pointwise convolutional layer. This CNN is an assemble of depth-wise separable convolutional layers with residual connections (as depicted in Figure 4). The depth-wise convolutional layer is used on every input data channel. Output channels are guided by pointwise convolutional layer (with dimensions of 1 × 1), with the help of depth-wise convolution to a new channel space.

### 2.4. DenseNet

**Figure 5:**
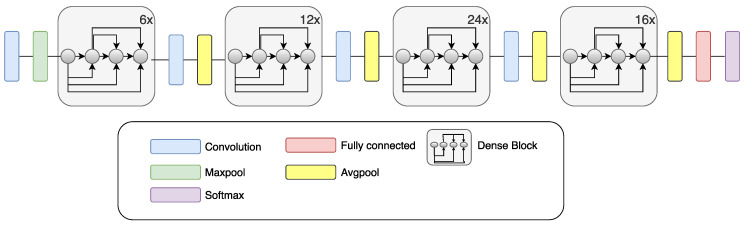
Schematic drawing of the DenseNet CNN [51].

Vanishing of the gradient is a network training problem which is connected with CNN depth. DenseNet, as well as ResNet, fights this problem. The architecture of the DenseNet is based on all layers’ connection, which provides the best information stream between layers [49]. In the DenseNet network structure, each layer receives inputs from all previous layers, and it connects its outputs to every layer ahead. The feature maps at each layer are serially chained to carry on data from previous layers to subsequent layers. Due to the reason that there is no need to learn redundant information, the number of parameters is decreased. All layers are connected, so the DenseNet efficiently preserves the learned information. DenseNet121, a specific implementation of the DenseNet used in this paper, exhibits excellent classification performance when it comes to small training datasets, and is not prone to overfitting [49]. Figure 5 shows a diagram of DenseNet.

### 2.5. Logistic Regression Classifier 

Logistic Regression is a supervised Machine Learning (ML) method used for classification tasks. The input X is a matrix containing *N* data instances, each described by *K* features. Inputs *x_ij_* are *K*-length feature-vectors (*x_i_*’*s*), which are continuous, with indexes j=1,……,K and i=1,……, N. Output *y_i_* falls in the interval {0,1} and it is a binary variable, with Bernoulli distribution and parameter *p_i_*. The decision/activation function of “Linear Classifier” Logistic Regression is called ‘logistic function’ (sigmoid). The main characteristic of the sigmoid function is to determine the output of one system in the interval (0,1), regardless of the variables on its input. The posteriors are expressed through the logistic function as

(1) $$P(Y/X)=\frac{1}{1+e^{-f\left(x\right)}},$$

In the above expression features $\left(x_{j}\right)\;$ and the corresponding weights $\left(\beta_{j}\right)$ make up the function  f(x).

In addition to the ‘logistic function,’ this Machine Learning (ML) method has an “objective function”—“Maximum Likelihood Estimation (MLE).” Its purpose is to make the “likelihood function” of the training process as large as possible and is expressed with

(2) $$\arg\underset{\beta }{\max}:\log\left\{\prod_{i=1}^{n}P{\left(y_{i}/x_{i}\right)}^{y_{i}}{\left(1-P\left(y_{i}/x_{i}\right)\right)}^{\left(1-y_{i}\right)}\right\},$$

Its parameters are: the output y which is in the interval (0,1), $P\left(y_{i}|x_{i}\right)$ is the posterior probability and is given with 1(1+e−f), and the weights vector in f(x)−β.

### 2.6. Support Vector Machine (SVM)

The SVM is a discriminative classifier formally defined by a separating hyperplane. Given a set of training data, the SVM finds an optimal hyperplane that categorizes new examples. Tuning hyperparameters of an SVM model consists of the kernel choice, impact of regularization, gamma, and margin. 

The kernel can be linear, polynomial, exponential, etc. The kernel type we considered in this work was linear. For the linear kernel, the prediction for input is calculated with

(3) $$f\left(x\right)=B\left(0\right)+sum\left(a_{i}*\left(x,x_{i}\right)\right).$$

It is obtained with the dot product of the input (*x*) and each of the support vectors (*x_i_*). It calculates the inner products of a new input vector (*x*) with all support vectors in training data. The coefficients B(0) and $a_{i}$ (for each input) are estimated from the training data by the learning algorithm.

The polynomial kernel and exponential kernel are given with

(4) $$K\left(x,x_{i}\right)=1+sum{\left(x*x_{i}\right)}^{\wedge }d$$

(5) $$K\left(x,x_{i}\right)=\exp(-gamma*sum\left(\left(x-x_{i}^{2}\right)\right)$$

The regularization parameter gives SVM optimization information to what extent to invalidate misclassification of every single training sample. The optimization with a large regularization parameter aims to achieve a better classification accuracy using a smaller-margin hyperplane. A small value of the regularization parameter will cause a larger-margin separating hyperplane, usually resulting in a reduced classification accuracy. The extent of influence of each training example is determined with the gamma parameter. Small values of gamma imply ‘far,’ and big values of gamma imply ‘close.’ 

### 2.7. Dimensionality Reduction 

The dimensions of the extracted features from the average pooling layers are one or two orders of magnitude smaller compared to features extracted from the convolutional layers. In our research, we wanted to have an even influence of the two types of extracted features on the classification process: the ones from the average pooling layers as well as from the convolutional layers. Additionally, we did not want the convolutional layer features to have a predominant effect on the classification accuracy because of the difference in vector dimensionality. This is the reason we performed a reduction of dimensionality of the convolutional layer features with Principal Component Analysis (PCA), before we concatenated them to average pooling layer features.

The PCA is a dimensionality reduction technique that determines a new group of data dimensions (a group of the basis of views), which are used for projecting the data from the original higher-dimensional space to a representation with smaller dimensions while preserving most of the variance in the data. All the new dimensions are independent because of their orthogonality, and are ranked depending on the variance of data within them. The first principal component preserves the most variance. The following steps can summarize the PCA:

1. Calculate the covariance matrix X of input data points with dimensions *m* × *n*;
2. Eigenvectors and corresponding eigenvalues should be calculated next;
3. Order the eigenvectors according to their eigen, such that they are decreasing;
4. New reduced *k* dimensions will be the first *k* eigenvectors;
5. Transform the original n-dimensional data points into k dimensions.

The new matrix has *n* data, points each of them with *k* features

(6) $${\left[new\;data\right]}_{k*n}={\left[top\;k\;eigenvectors\right]}_{k*m}{\left[original\;data\right]}_{m*n}$$

The purpose of PCA is to spread out data to have a high variance, along with a smaller number of dimensions, and there should be no covariance between dimensions. Therefore, the covariance matrix of transformed data points should be diagonal. 

### 2.8. Datasets

#### 2.8.1. UC-Merced Dataset 

**Figure 6:**
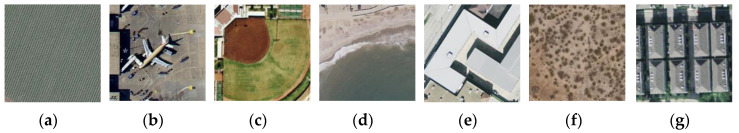

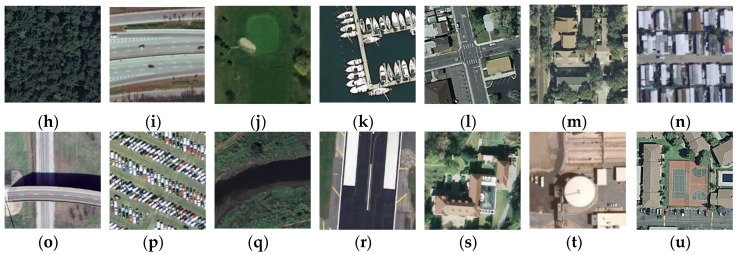
Image classes in UC-Merced dataset: (**a**) agriculture; (**b**) airplane; (**c**) baseball diamond; (**d**) beach; (**e**) buildings; (**f**) chaparral; (**g**) dense residential; (**h**) forest; (**i**) freeway; (**j**) golf course; (**k**) harbor; (**l**) intersection; (**m**) medium residential; (**n**) mobile home park; (**o**) overpass; (**p**) parking lot; (**q**) river; (**r**) runway; (**s**) sparse residential; (**t**) storage tanks; and (**u**) tennis court.

The UC-Merced dataset [21] has 21 classes of aerial scene remote sensing images, as can be seen in Figure 6. The pixel resolution of images is one foot. They are cropped to regions of 256 × 256 pixels. The original images were downloaded from the United States Geological Survey (USGS) National Map of the following US regions: Birmingham, Boston, Buffalo, Columbus, Dallas, Harrisburg, Houston, Jacksonville, Las Vegas, Los Angeles, Miami, Napa, New York, Reno, San Diego, Santa Barbara, Seattle, Tampa, Tucson, and Ventura. Each of the 21 classes has 100 images, which are manually selected and uniformly labeled: agricultural, airplane, baseball diamond, beach, buildings, chaparral, dense residential, forest, freeway, golf course, harbor, intersection, medium-density residential, mobile home park, overpass, parking lot, river, runway, sparse residential, storage tanks, and tennis courts. What makes the dataset difficult for classification is that there are some classes with similar object shapes and distribution, e.g., dense residential, medium residential, and sparse residential. The difference between these classes is mostly in object density.

#### 2.8.2. WHU-RS Dataset

**Figure 7:**
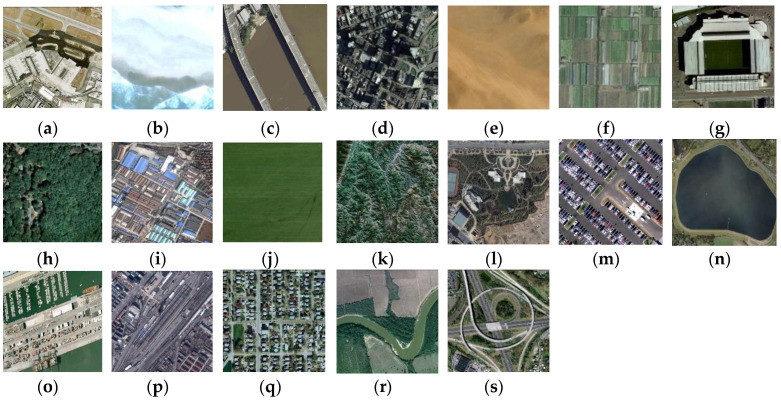
Image classes in WHU-RS dataset: (**a**) airport; (**b**) beach; (**c**) bridge; (**d**) commercial; (**e**) desert; (**f**) farmland; (**g**) football field; (**h**) forest; (**i**) industrial; (**j**) meadow; (**k**) mountain; (**l**) park; (**m**) parking; (**n**) pond; (**o**) port; (**p**) railway station; (**q**) residential; (**r**) river; and (**s**) viaduct.

The WHU-RS dataset [41] is collected from Google Earth imagery. There are 1005 images assigned to 19 classes. The images are of high spatial resolution, having 600 × 600 pixels. At least 50 images represent each class. Sample images of each class are presented in Figure 7. The images in this dataset represent aerial scenes from different places all over the Earth. So far, the WHU-RS dataset has been used extensively in research studies of different aerial scene classification methods.

### 2.9. Experimental Setup

For all experiments described below, we used moderate data augmentation on the training set, creating five patches from a single image, by applying translations, rotations, changes in scale, shearing, and horizontal and vertical flips of the images of remote sensing dataset. Aggressive data augmentation can produce even more patches from the original image.

We conducted our simulations in two directions. First, we extracted features from three separate layers of each of the CNNs: ResNet50, InceptionV3, Xception, and DenseNet121. The CNNs were pre-trained, and the parameters of the original deep architectures were kept. In the ResNet50, the following layers were used: average pooling layer, the last convolutional layer before the average, and bn4f_branch2c layer. In the InceptionV3, the following layers were used: average pooling layer, mixed_10 layer, and mixed_8 layer. In the Xception, the following layers were used: average pooling layer, block14_sepconv2_act, and block14_sepconv1_act layer. Finally, for the DenseNet121, the following layers were used: average pooling layer, the last convolutional layer before the average, and conv4_block24_concat. The input image size for ResNet50 and the DenseNet121 was 224 × 224, and the input image size for InceptionV3 and the Xception was 229 × 299. The feature extraction was performed for two datasets: the UC-Merced and WHU-RS dataset. Training/test dataset ratio was 80% vs. 20% for the UC-Merced dataset, and 60% vs. 40% for the WHU-RS dataset. The picked out split ratios are the same as the ones chosen in the related work we compare our approaches to. The splits are random. There was no stratification used. We did not want to stratify train/test data splits to provide a completely random process. Controlled data splits (with an equal number of each image class in every train/ test split) may influence the classification accuracy and lead to the higher mean value and lower standard deviation of the results. Still, the purpose was not to avoid the worst classification results. Every input image was pre-processed according to the requirements of the appropriate CNN. Moderate data augmentation was used on the training set (image data generator produced five patches per image): images were rotated, shifted, sheared, zoomed, and horizontally flipped. After the features were extracted, a linear classifier was trained (LRC or SVM). We performed a grid search in order to tune model hyperparameters (regularization parameter C). This first part of the survey was conducted with the sole aim of gaining insight into which CNN layer leads to the best classification results. Here, it is essential to mention that the average pooling layer or the last convolutional layer does not always give the best classification results. In some of the networks, the best results were obtained using the inner (intermediate) convolutional layer.

The second part of our survey study was conveyed for boosting the classification performance using feature fusion. This time, the features were extracted from two different layers of two different CNNs, in a way that one layer was always the average pooling layer, and the other was the convolutional layer (the last one or an intermediate convolutional layer). The combinations of layers and networks were determined from the results obtained in the first part of the simulations. Here, the images were also appropriately resized, pre-processed, and moderate data augmentation was again applied to the training set: images were rotated, shifted, sheared, zoomed, and horizontally flipped. Training/ test dataset ratio was 80%/20% and 50%/50% for the UC-Merced dataset, and 60%/40% and 40%/60% for the WHU-RS dataset. The splits are random and without stratification again. Before the feature fusion (concatenation), the PCA transformation is performed on features extracted from the convolutional layer. Next, the L2 normalization is done on the features extracted from the average pooling layer, and PCA transformed features, and, finally, the features are fused. SVM is used for the classification task. The classification results obtained with this method are comparable to state-of-the-art methods based on feature extraction.

All simulations were performed on OS Ubuntu 18.04 with Keras v2.2.4. Google’s library TensorFlow v1.12.0 [53], was backend to Keras. The hardware setup was: CPU i7-8700 3.2 GHz and 64 GB RAM. The graphical processor unit was Nvidia GeForce GTX 1080 Ti, with 11 GB of memory and CUDA v9.0 installed on it.

### 2.10. Evaluation Metrics

In this article, we use two evaluation metrics: Overall Accuracy OA (equivalent to classification accuracy) and a confusion matrix. These two types of metrics are usual for analysis of results and comparison with similar cutting-edge methods in classification tasks articles. The classification accuracy is the ratio between the number of adequately classified test images (from all image classes) and the total number of test images. The value of this metric is less than 1. Despite overall accuracy, the classification accuracy on each separate image class is presented in a confusion matrix. The confusion matrix is a graphical display (table) of the classification accuracy on each class of the dataset. This table clearly shows the errors of each separate class and confusion between different classes. In a confusion matrix, the columns represent the predicted classes, and the rows represent real classes. In a normalized confusion matrix, the item *x_ij_* is the percentage of images that are classified as they belonged to the i-th class, but their real class is j-th [54]. Ideally, an accurate prediction model leads to a diagonal confusion matrix, or with high values on the diagonal and shallow values in other entries. In our experimental setup, the dataset was divided into training and testing sets. The division was random, without stratification, and it was made according to the scales listed in the previous section. To check the reliability of the results, all cases where the biggest OA is obtained are repeated ten times. After that, the mean value and the standard deviation (SD) from each experiment are calculated. 

## 3. Results

### 3.1. Classification Founded on Extracted Features from Different CNN Layers

As mentioned in the experimental setup, the first part of the simulations is devoted to classification based on features extracted solely from one network layer. The obtained results are displayed in Table 1, with 80% of UC-Merced dataset used as a training set, and in Table 2 for the WHU-RS dataset under the training ratio of 60%. The remaining instances were used in the test set, and no instances were left out of the analysis. Experiments were extensive across all four pre-trained CNNs, and three different layers in each of them. Extracted features were fed to two different linear classifiers: LRC and SVM. 

As it is known, the average pooling layer is a replacement for the fully connected layers in the architecture of a CNN. Therefore we expected that the features extracted from that layer would lead to the highest classification accuracies. On the other hand, convolutional layers, especially the intermediate ones, give features which represent mid-level information (e.g., object parts), but not the spatial dependencies between them (e.g., the whole object), and the classification accuracies should deteriorate compared to the features extracted from average pooling layers.

However, if we carefully analyze Table 1 and Table 2, we can conclude for both datasets, as well as for both linear classifiers, that the best accuracies for each of the pre-trained CNNs, except for ResNet50, are obtained with features extracted from the intermediate convolutional layer. That is the mixed_8 layer for the InceptionV3, block14_sepconv1_act layer for the Xception, and conv4_block24_concat layer for the DenseNet121. These results gave us directions for the second part of our experiments. Namely, we extracted features from convolutional layers of interest, then PCA transformed them and fused them with features extracted from the average pooling layer of different CNN. With this method, we aimed to boost the classification accuracy on the UC-Merced and the WHU-RS dataset.

### 3.2. Classification Based on Features Fusion with PCA Transformation

The second part of the simulations presents the capacity of the evaluated method: the linear classification of features fused from average pooling layer and PCA transformed features from some of the convolutional layers. In the PCA decomposition, we used 2010 components, which provided a good balance between performance and dimensionality reduction. For the Linear SVM, we used a grid search approach [55] to select the value for C from the set of values: 0.0001, 0.001, 0.01, 0.1, 1, 10, 100, 1000, 10,000, and 100,000. The maximum iterations were 5000, using 3-fold standard cross-validation using the training subset. The default SVM parameters were significantly worse without the grid search.

The research was done for different combinations of layers, and the SVM as a linear classifier for the UC-Merced dataset, and is presented in Table 3. To evaluate the classification accuracy of the examined technique, we compared it to the achieved classification accuracy of a couple of similar cutting-edge classification techniques on the UC Merced Land-Use dataset. The performance of different techniques is shown in Table 4, which is a common way of reporting results using these methods, as in [56]. As Table 4 shows, the proposed method for feature fusion with PCA transformation gives a classification accuracy comparable to the competitive methods. Methods that outperform our architecture can be found: the integration of a global context features and features concerning local objects GCFs+LOFs [57] and Inception-v3-CapsNet [54]. In order to check the reliability of the results, all cases where the largest OA is obtained are repeated ten times, and the mean and standard deviation on the testing sets are calculated, as shown in Table 5. It has to be noted that the averaged accuracies are somewhat lower than the ones shown in Table 4.

**Figure 8:**
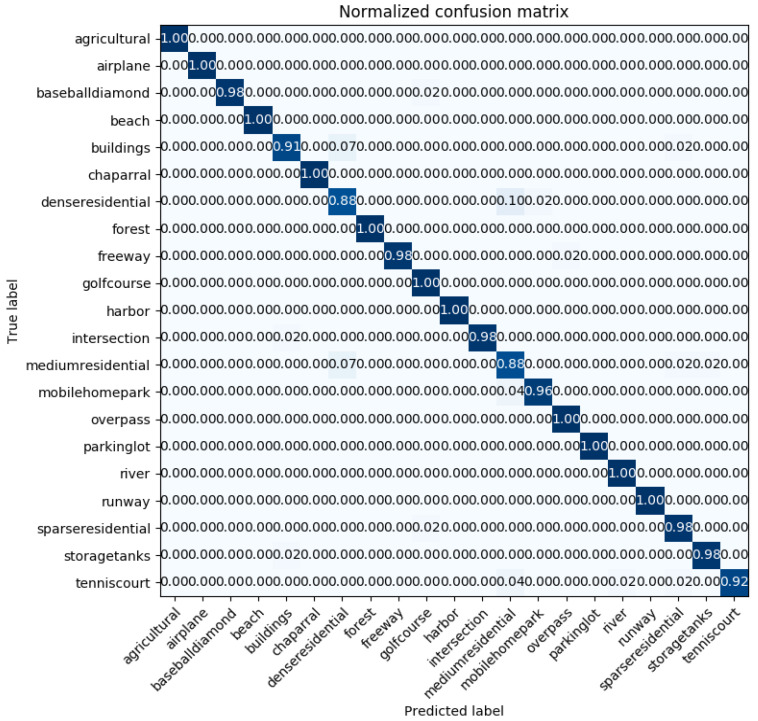
The confusion matrix of the examined method, with 50% of the UC-Merced dataset as a training set for InceptionV3 mixed_8 (PCA) + Xception average pooling. The highlighting of the confusion matrix is a heat map, where white represents 0s, and dark blue represents 1s.

**Figure 9:**
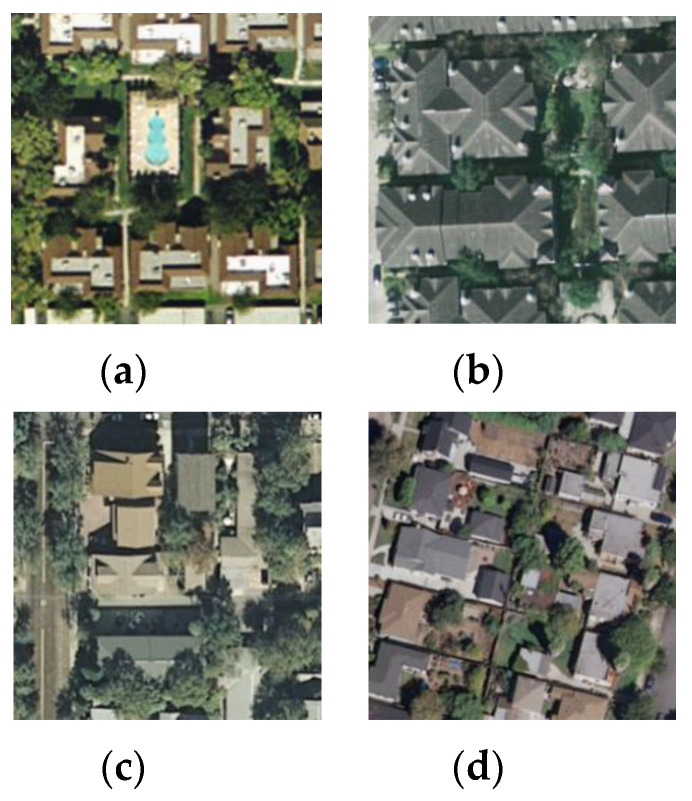
Class representatives of the UC Merced LandUse dataset: (**a**) dense residential; (**b**) dense residential; (**c**) medium residential; (**d**) medium residential.

Figure 8 displays the confusion matrix plotted in the case of the best classification accuracy obtained by the InceptionV3 mixed_8 (PCA) + Xception average pooling layer with 50% of the UC-Merced dataset as a training set. The confusion matrix shows that the worst classified categories are ‘dense residential’ and ‘medium residential.’ Those two classes are easily confused with each other. This comes from the fact that the ‘dense residential’ and ‘medium residential’ classes have similar image structures, for example, the building shapes and distribution, and it is difficult to differentiate them from each other, which can be seen in Figure 9. However, it is noticeable that ‘dense residential’, as well as ‘medium residential’, achieved an accuracy of 88%. This accuracy outperformed the GCFs+LOFs [57], with a ‘dense residential’ accuracy of 74%, and the Inception-v3-CapsNet [54], with a ‘dense residential’ accuracy of 80%. These two methods are the best ones ranked in Table 4.

**Figure 10:**
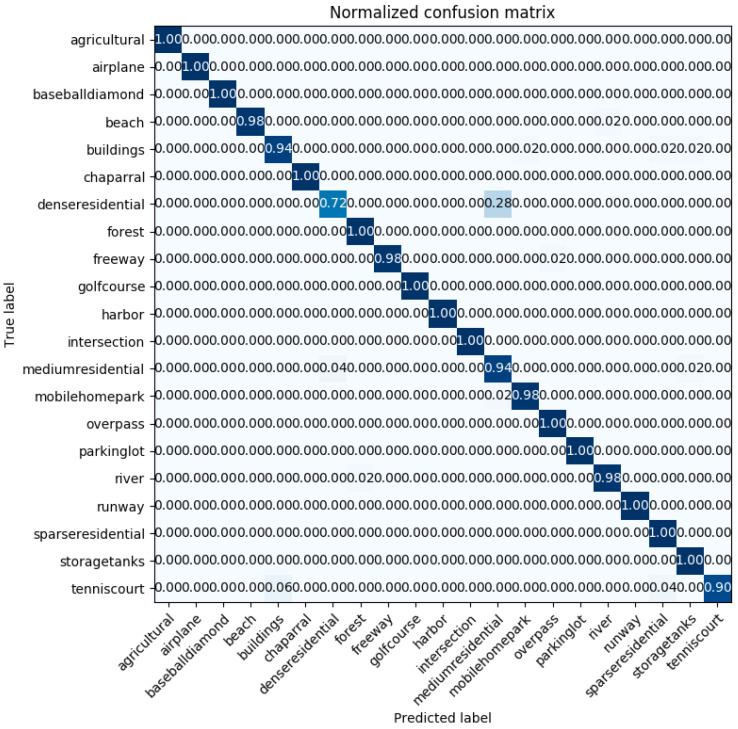
The confusion matrix of the examined method, with 50% of the UC-Merced dataset as a training set for InceptionV3 mixed_8 (PCA) + ResNet50 average pooling. The highlighting of the confusion matrix is a heat map, where white represents 0s, and dark blue represents 1s.

Figure 10 displays the confusion matrix plotted for the achieved classification accuracy by the InceptionV3 mixed_8 (PCA) + ResNet50 average pooling with 50% of the UC-Merced dataset as a training set. 

The second part of the research was repeated for the WHU-RS dataset as well: the linear classification of features fused from average pooling layer(s) and PCA transformed features from some of the convolutional layers. The classification accuracy of different combinations of layers, and the SVM as a linear classifier, is presented in Table 6. To evaluate the classification accuracy of the examined technique, we compared it to the achieved classification accuracy of a couple of state-of-the-art classification methods on the WHU-RS dataset, as displayed in Table 7. As it is depicted in Table 7, the examined architecture for feature fusion with PCA transformation gives a classification accuracy comparable to the state-of-the-art methods. It can be noted that our proposed method for a training ratio of 40% outperforms all the other cutting-edge classification methods. To check the reliability of results, all cases where the largest OA is obtained are repeated ten times on testing sets. Afterward, we calculated the mean value and standard deviation on the achieved results, as shown in Table 8. 

**Figure 11:**
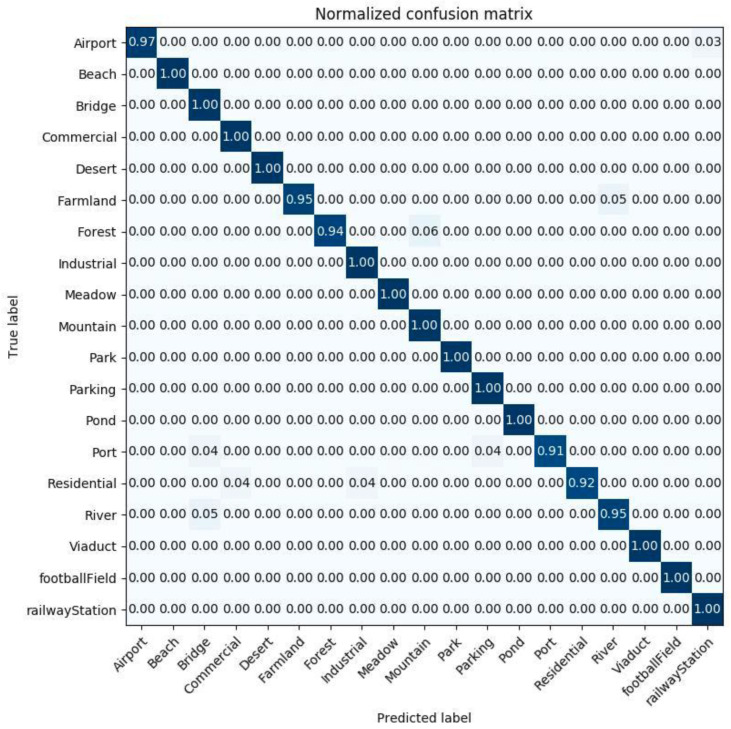
The confusion matrix of the examined method, with 60% of WHU-RS dataset as a training set for InceptionV3 mixed_8 (PCA) + ResNet50 average pooling.

**Figure 12:**
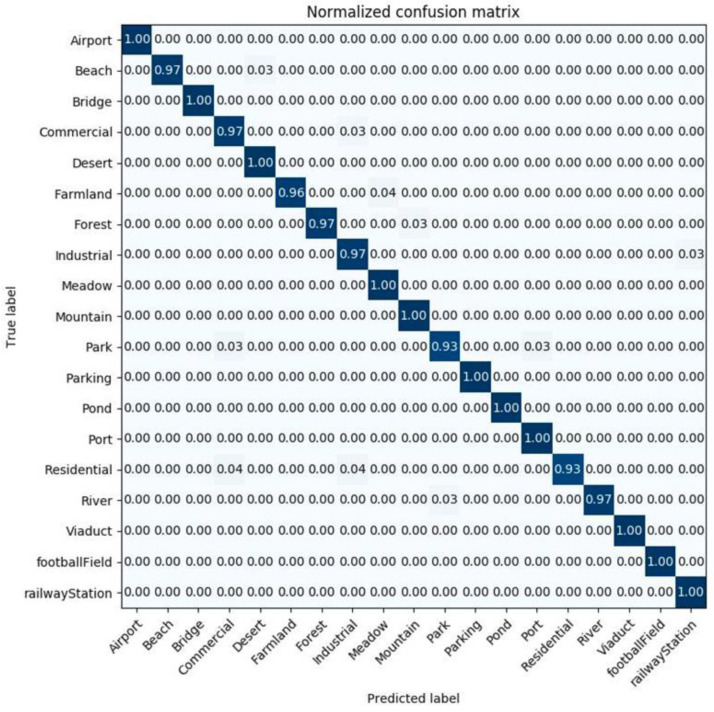
The confusion matrix of the examined method, with 40% of WHU-RS dataset as a training set for DenceNet121 conv5_block16_concat (PCA) + ResNet50 average pooling.

Figure 11 and Figure 12 show the confusion matrices generated from the classification result by the InceptionV3 mixed_8 (PCA) + ResNet50 average pooling with the training ratio of 60%, and by DenseNet121 conv5_block16_concat (PCA) + ResNet50 average pooling, for a training ratio of 40%.

## 4. Discussion

We are aware that there a lot of powerful techniques for remote sensing datasets classification, which can achieve astonishing accuracies. The aim of our research was not to outperform every one of them. The purpose of this article is to contribute in the directions that were not researched intensively, for example the specific types of pre-trained CNNs as feature extractors. From the finished simulations and obtain results, the following valuable concepts can be summarized:

- Despite many research studies and theories that fully connected layers (average pooling layers) give features that achieve the biggest classification accuracies, this study suggests the opposite. In remote sensing dataset classification through transfer learning with feature extraction, the biggest accuracies are achieved by features extracted from the intermediate convolutional layers. It is a result of the fact that CNNs used for feature extraction are originally trained on quite different datasets, and that the features of the average pooling layers are connected with the semantic meaning of the images. On the other hand, features extracted from the intermediate convolutional layers represent some basic image elements like lines, edges, colors, shadows, etc., and are common for a variety of datasets. Special attention should be dedicated to the mixed_8 convolutional layer of InceptionV3 pre-trained CNN.
- The classification of aerial scenes conducted with two linear classifiers: LRC and SVM gave similar results and thus confirmed our conclusions. When features were extracted solely from one layer (regardless of whether it was extracted from an average pooling layer or some of the convolutional layers), the trends of increasing or decreasing the classification accuracies were the same with any of the classifiers. For all pre-trained CNNs, except for ResNet50, the accuracies increased by moving to intermediate layers. It is a challenging task to move further to lower layers and to compare the classification accuracies.
- The proposed method for remote sensing image datasets classification, based on a fusion of features with PCA transformation, leads to accuracies which are comparable to the state-of-the-art methods. However, even though there are methods attaining higher classification accuracies on the UC-Merced dataset, the classification accuracy attained on the class “dense residential” (the most demanding one) is higher, compared to the other classification methods presented in the literature. Namely, our method achieved an accuracy of 0.88 under the training ratio of 50% on the UC-Merced dataset, compared to the GCFs +LOFs with a “dense residential” accuracy of 0.74 and the Inception-v3-CapsNet with “dense residential” accuracy of 0.8. This can find its usage when it is about the classification of datasets with image classes with big inter-class similarities (image classes which are easy to confuse with each other).
- Our method of feature fusion with PCA transformation is competitive to the cutting-edge methods. However, it should be noted that it performs better under a smaller percentage of the training set. Under the training ratio of 40% on the WHU-RS dataset, it gives the classification accuracy of 98.26 ± 0.40, which outperforms other methods in the literature. This can be useful in situations when there is no possibility for time-excessive training of the classifiers, and the acceptable classification accuracy can be achieved with a smaller ratio of the training set. Time-excessive training is never a good idea, especially when there is a need for repetitive experiments.
- In our simulations, moderate data augmentation on the training set is used, which certainly helped to achieve good classification results. Data augmentation can be more aggressive, and can produce more patches from the original image (here, data augmentation gave five patches from a single image). It can be done by translations, rotations, changes in scale, shearing, and horizontal and vertical flips of the images of remote sensing dataset. This can lead to improving the classification accuracy, as the result of the larger amount of data to train the linear classifiers, which can be even more competitive.
- In the performed experiments, the dataset split is completely random; in other words, no stratification is used. A stratified data split may lead to bigger classification accuracies, because image classes which are more difficult to differentiate are represented equally with all the other classes. This choice is made on purpose (the process should be random), even it brings a lower mean value and a bigger standard deviation.

The above observations give us valuable information and directions for researching more competitive methods on bigger and more complex datasets to provide the progress in remote sensing image classification.

As an alternative, we attempted to use the features extracted from the deep learning models, and then perform traditional feature selection and classification with other ensemble-based algorithms, such as Random Forest, XGBoost, Adaboost, or Extremely Randomized Trees. However, all datasets exported after the feature extraction were about 550 GB, so we were not able to effectively perform feature extraction based on ranking or correlation. Wrapper and hybrid methods were even less suitable, because of their complexity. Be that as it may, we believe that such analysis is worthwhile, and could be valuable for improving the classification performance. 

## 5. Conclusions

In our paper, the two-stream concatenation method for the classification of remote sensing high-resolution images was used. In our experiments, at first, CNNs pre-trained on ImageNet dataset were used to extract features from the original aerial image from different layers of neural networks’ architecture. After the extraction, the features extracted from the average pooling layer and the PCA transformed features from a convolutional layer were concatenated, to form a unique representation of features. In the end, we used the SVM classifier for the classification of the final set of features. We tested our architecture on two datasets. In comparison with other state-of-the-art methods, our architecture achieved comparable results. The proposed method can be relevant when there is a need to perform training of a classifier with a small ratio on the training dataset. Additionally, this technique might be a good solution for the classification of datasets with image classes with big inter-class similarities, like “dense residential” of the UC-Merced dataset.

The proposed technique for remote sensing image classification can be further explored with extracting features from lower layers of pre-trained deep CNN. In addition to this, stratification can also have an impact on the classification accuracy of the researched technique. So, to boost the accuracy and to come closer to the best classification practices, the spilt of the training/testing dataset should be stratified. All gathered knowledge and experience could be checked on other remote sensing datasets, not necessarily publicly available, preferably small scale, because the proposed classification method gives good results with a small portion of the training set.

## Figures and Tables

**Table 1:** The classification accuracy (OA (%)) of linear classification with a Logistic Regression Classifier (LRC) and a Support Vector Machine (SVM), using features extracted from different layers with 80% of UC-Merced dataset as a training set.

Method	LRC	SVM
**ResNet50**		
avg pooling	96.19	95.71
last conv layer	95.71	97.38
bn4f_branch2c	94.52	93.57
**InceptionV3**		
avg pooling	96.67	95
mixed_10	95.48	95.71
mixed_8	98.10	98.33
**Xception**		
avg pooling	93.57	94.76
block14_sepconv2_act	93.81	94.29
block14_sepconv1_act	96.43	95.71
**DenseNet121**		
avg pooling	95.48	93.81
conv5_block16_concat	96.67	94.05
conv4_block24_concat	97.14	95.24

**Table 2:** The classification accuracy (OA (%)) of linear classification with LRC and SVM of features extracted from different layers with 60% of WHU-RS dataset as a training set.

Method	LRC	SVM
**ResNet50**		
avg pooling	98.01	97.01
last conv layer	98.01	97.76
bn4f_branch2c	95.52	96.02
**InceptionV3**		
avg pooling	95.78	95.02
mixed_10	94.53	95.52
mixed_8	97.26	97.26
**Xception**		
avg pooling	93.28	93.53
block14_sepconv2_act	94.28	94.53
block14_sepconv1_act	95.27	95.52
**DenseNet121**		
avg pooling	96.52	95.27
conv5_block16_concat	96.27	95.52
conv4_block24_concat	96.27	96.27

**Table 3:** The classification accuracy (OA (%)) of the linear classification of fused features with Principal Component Analysis (PCA) transformation, with 80% and 50% of UC-Merced dataset as a training set.

Method	80% of UCM Dataset as a Training Set	50% of UCM Dataset as a Training Set
ResNet50 last conv layer (PCA) + InceptionV3 avg pooling	97.14	97.33
ResNet50 last conv layer (PCA) + Xception avg pooling	97.62	97.43
DenseNet121 conv5_block16_concat (PCA) + Xception avg pooling	97.86	96.67
DenseNet121 conv4_block24_concat (PCA) + Xception avg pooling	97.86	96.57
InceptionV3 mixed_10 (PCA) + ResNet50 avg pooling	97.62	96.57
InceptionV3 mixed_8 (PCA) + ResNet50 avg pooling	98.33	97.43
InceptionV3 mixed_10 (PCA) + Xception avg pooling	95.95	95.14
InceptionV3 mixed_8 (PCA) + Xception avg pooling	98.57	97.62
DenseNet121 conv5_block16_concat (PCA) + ResNet50 avg pooling	97.14	96.67
DenseNet121 conv4_block24_concat (PCA) + ResNet50 avg pooling	96.9	95.24
Xception block14_sepconv2_act (PCA) + DenseNet121 avg pooling	96.67	96.48
Xception block14_sepconv1_act (PCA) + DenseNet121 avg pooling	98.57	96.29

**Table 4:** The classification accuracy (OA (%) and SD) of the examined method and the reference methods, with 80% and 50% of UC-Merced dataset as a training set.

Method	80% of UCM Dataset as a Training Set	50% of UCM Dataset as a Training Set
CaffeNet [44]	95.02 ± 0.81	93.98 ± 0.67
GoogLeNet [44]	94.31 ± 0.89	92.70 ± 0.60
VGG-16 [44]	95.21 ± 1.20	94.14 ± 0.69
SRSCNN [58]	95.57	/
CNN-ELM [59]	95.62	/
salM^3^LBP-CLM [60]	95.75 ± 0.80	94.21 ± 0.75
TEX-Net-LF [61]	96.62 ± 0.49	95.89 ± 0.37
LGFBOVW [62]	96.88 ± 1.32	/
Fine-tuned GoogLeNet [63]	97.10	/
Fusion by addition [64]	97.42 ± 1.79	/
CCP-net [65]	97.52 ± 0.97	/
Two-stream Fusion [50]	98.02 ± 1.03	96.97 ± 0.75
DSFATN [66]	98.25	/
Deep CNN Transfer [37]	98.49	/
InceptionV3 mixed_8 (PCA) + Xception avg pooling (Ours)	98.57	97.62
GCFs+LOFs [57]	99 ± 0.35	97.37 ± 0.44
Inception-v3-CapsNet [54]	99.05 ± 0.24	97.59 ± 0.16

**Table 5:** The overall accuracy (%) and standard deviation of the examined method, with 80% and 50% of the UC-Merced dataset as a training set.

Method	80% of UCM Dataset as a Training Set	50% of UCM Dataset as a Training Set
InceptionV3 mixed_8 (PCA) + ResNet50 avg pooling	97.67 ± 0.64	97.00 ± 0.65
InceptionV3 mixed_8 (PCA) + Xception avg pooling	97.86 ± 0.59	96.6 ± 0.65

**Table 6:** The classification accuracy (OA (%)) of the linear classification of fused features with PCA transformation, with 60% and 40% of WHU-RS dataset as a training set.

Method	60% of WHU-RS Dataset as a Training Set	40% of WHU-RS Dataset as a Training Set
ResNet50 last conv layer (PCA) + InceptionV3 avg pooling	98.26	95.02
ResNet50 last conv layer (PCA) + Xception avg pooling	97.62	96.52
DenseNet121 conv5_block16_concat (PCA) + Xception avg pooling	97.01	95.69
DenseNet121 conv4_block24_concat (PCA) + Xception avg pooling	97.76	96.68
InceptionV3 mixed_10 (PCA) + ResNet50 avg pooling	96.27	95.85
InceptionV3 mixed_8 (PCA) + ResNet50 avg pooling	98.01	98.67
InceptionV3 mixed_10 (PCA) + Xception avg pooling	96.77	96.02
InceptionV3 mixed_8 (PCA) + Xception avg pooling	98.01	96.35
DenseNet121 conv5_block16_concat (PCA) + ResNet50 avg pooling	98.76	98.34
DenseNet121 conv4_block24_concat (PCA) + ResNet50 avg pooling	96.77	96.52
Xception block14_sepconv2_act (PCA) + DenseNet121 avg pooling	97.51	96.35
Xception block14_sepconv1_act (PCA) + DenseNet121 avg pooling	97.76	96.52
DenseNet121 conv5_block16_concat (PCA) + InceptionV3 avg pooling	96.27	97.51
DenseNet121 conv4_block24_concat (PCA) + InceptionV3 avg pooling	98.01	97.18

**Table 7:** The classification accuracy (OA (%) and SD) of the examined method and the reference methods, with 60% and 40% of WHU-RS dataset as a training set.

Method	60% of WHU-RS Dataset as a Training Set	40% of WHU-RS Dataset as a Training Set
Bag of SIFT [29]	85.52 ± 1.23	/
MS-CLBP + BoVW [67]	89.29 ± 1.30	/
GoogLeNet [44]	94.71 ± 1.33	93.12 ± 0.82
VGG-VD-16 [44]	96.05 ± 0.91	95.44 ± 0.60
CaffeNet [44]	96.24 ± 0.56	95.11 ± 1.20
salM^3^LBP-CLM [60]	96.38 ± 0.82	95.35 ± 0.76
TEX-Net-LF 61]	96.62 ± 0.49	95.89 ± 0.37
InceptionV3 mixed_8 (PCA) + ResNet50 avg pooling (Ours)	98.13 ± 0.51	/
DCA by addition [64]	98.70 ± 0.22	97.61 ± 0.36
Fusion with saliency detection [50]	98.92 ± 0.52	98.23 ± 0.56
DenseNet121 conv5_block16_concat (PCA) + ResNet50 avg pooling (Ours)	/	98.26 ± 0.40

**Table 8:** The classification accuracy (OA (%) and SD) of the examined method, with 60% and 40% of the WHU-RS dataset as a training set.

Method	60% of WHU-RS Dataset as a Training Set	40% of WHU-RS Dataset as a Training Set
InceptionV3 mixed_8 (PCA) + ResNet50 avg pooling	98.13 ± 0.51	97.84 ± 0.53
DenseNet121 conv5_block16_concat (PCA) + ResNet50 avg pooling	98.01 ± 0.68	98.26 ± 0.40
